# Age at menarche and risks of gestational diabetes mellitus: a meta-analysis of prospective studies

**DOI:** 10.18632/oncotarget.23658

**Published:** 2017-12-23

**Authors:** Ya Xiao, Ruixue Chen, Minghao Chen, Anling Luo, Dayi Chen, Qiuer Liang, Yunfei Cai, Liguo Chen, Xiaoshan Zhao

**Affiliations:** ^1^ School of Traditional Chinese Medicine, Jinan University, Guangzhou, 510632, China; ^2^ Reproductive center, Guangdong Women and Children Hospital, Guangzhou, 511400, China; ^3^ School of Basic Medicine, Jinan University, Guangzhou, 510632, China; ^4^ School of Traditional Chinese Medicine, Southern Medical University, Guangzhou, 510515, China

**Keywords:** menarche, menarcheal age, gestational diabetes

## Abstract

**Background:**

Accumulating evidence suggests that early menarche is associated with adult obesity, which in turn may increase the risk of insulin resistance and hyperglycemia. However, the relation of menarcheal age with gestational diabetes mellitus (GDM) remains inconsistent across studies. The objective of this meta-analysis was to evaluate the association between age at menarche and GDM risk.

**Materials and Methods:**

We searched Medline (PubMed), Embase, Web of Knowledge and the Cochrane library through the end of May 2017. We pooled summary relative risks (RR) with 95% confidence intervals (CIs). Stata 12.0 software was used to analyse the data.

**Results:**

Five prospective studies were eligible for inclusion. The results of meta-analysis showed that women in the early menarcheal age group (at < 12 years of age) had a higher risk of GDM compared with those in the “not early” menarcheal age group (at ≥ 13 years of age) (pooled RR = 1.31, 95% CI: 1.05, 1.56) with moderate heterogeneity (I^2^ = 47.5%, *P* = 0.107). However, there was no obvious protection of late menarche (at ≥ 15 years of age) versus median menarche (at 13 years of age) (pooled RR = 1.12, 95% CI: 0.92, 1.32; I^2^ = 0%).

**Conclusions:**

The findings support an association between earlier age at menarche and increased risk of GDM. Age at menarche may help identify women with increased risk of developing GDM. However, considering the potential limitations in this study, further larger prospective studies are warranted to verify our findings.

## INTRODUCTION

Gestational diabetes mellitus (GDM) is one of the most common metabolic diseases during pregnancy, affecting approximately 1–14% of pregnant women [1]. GDM not only has adverse impact on maternal and child health but also significantly increases the medical costs [2]. Therefore, identifying the risk factors for GDM is of great importance to provide pregnant women with early prevention.

Menarche, the onset of first menstruation, is a landmark of puberty in girls. Age at menarche is declining [3], which is coincide with the trend of increasing prevalence of overweight/obesity in childhood and adolescent period [4, 5]. Studies suggested that early menarche increased the risk of type 2 diabetes (T2DM) [6], metabolic syndrome [7], all-cause or cardiovascular disease mortality [8, 9] and breast cancer [10]. However, potential relationships of menarche age and risk of GDM have not been clarified. Current evidence on the association between menarche and GDM is inconsistent. To our knowledge, no systematic review and meta-analysis has yet been performed on this topic. Therefore, the aim of the present study was to systematically review and examine the association between menarcheal age and the risk of GDM.

## RESULTS

**Figure 1:**
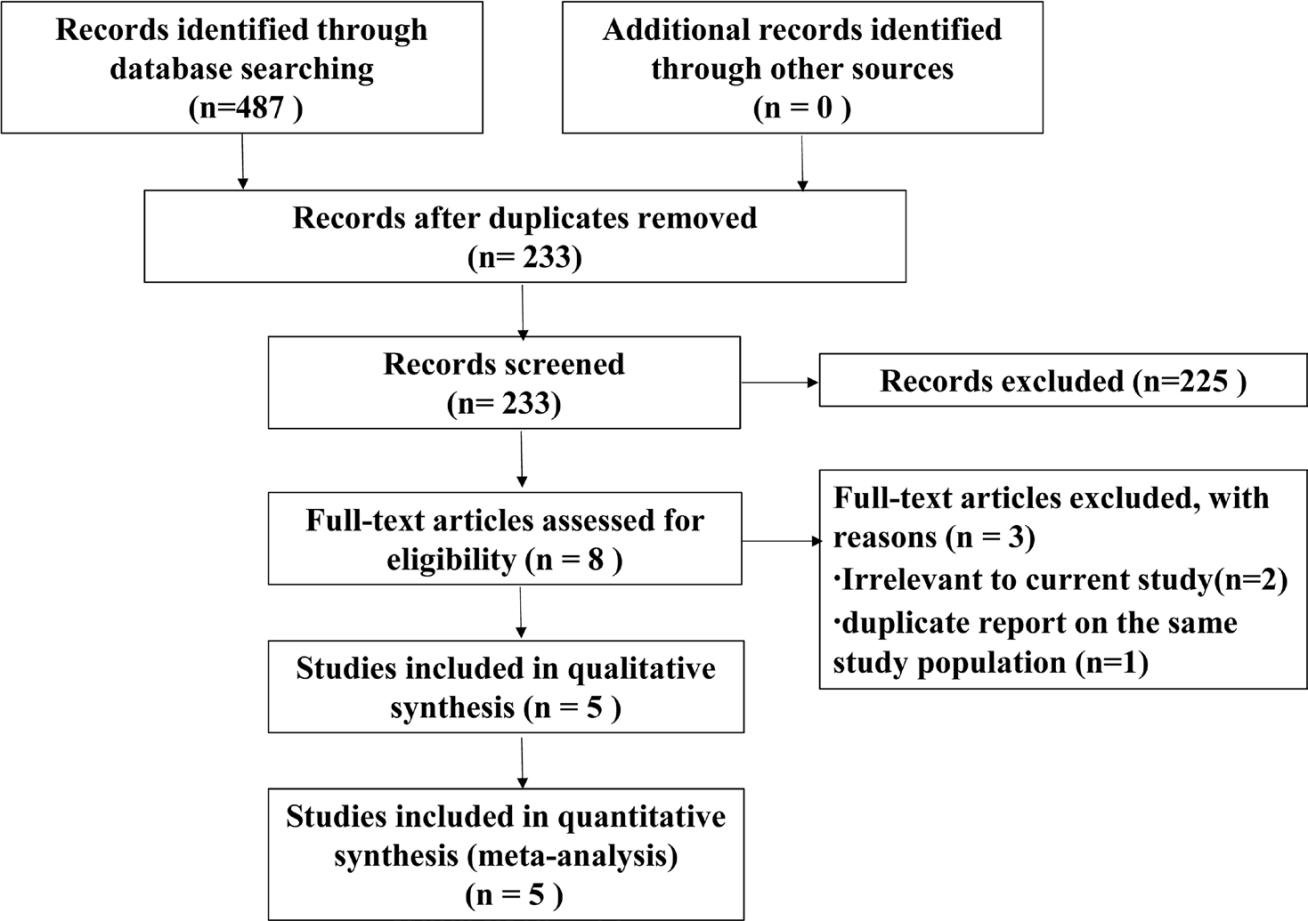
Flow diagram of study selection process

A total of 487 publications were identified by electronic search. Of the 487 articles, 254 were duplicates. The remaining 233 reports were retrieved, of which 225 were excluded on review of the titles and abstracts, leaving 8 articles for further evaluation. Finally, a total of 5 studies were included in this meta- analysis (Figure 1).

### Study characteristics

Characteristics of the included articles are shown in Table 1. The 5 included studies [11–15] were all cohort studies, with sample sizes ranged from 3,490 to 27,482 women. Of these prospective studies, three were conducted in the United States [11, 12, 15], one in Australia [14] and one in China [13]. In all studies, age at menarche was self-reported and grouped into 5 categories, with the exception of two studies [11, 15]. The duration of follow-up was longer than 10 years in three studies [11, 12, 14]. These studies were published between 2011 and 2017. A wide range of potential confounders considered by most studies included age, BMI, physical activity, smoking status and familial history of diabetes. Two studies identified GDM with oral glucose tolerance test [12, 13] and three studies based on self-reported diagnosis of GDM [11, 14, 15].

**Table 1:** Characteristics of Included Studies of Menarcheal Age and GDM Risk

Author, Publication Year, Country	Cases/Subject	Follow- up Period	Menarcheal Age Categories (Exposure Assessment)	RR (95% CI)	Matched/Adjusted Factors
Chen et al., 2016, USA (11)	at least 1058/27482	1989–2001	≥ 14 ≤ 11 12 13	1.0 (ref) 1.34 (1.14, 1.58) 1.13 (0.97, 1.31) 1.11 (0.95, 1.29)	Age, family history of diabetes, race/ethnicity, birth weight, somatotype at age 5 years, somatotype at age 10 years, alcohol consumption, smoking status, Alternate Healthy Eating Index 2010 (quintiles), total physical activity, marital status, and oral contraceptive use
Dishi et al., 2011, USA (12)	185/3490	1996–2008	13 ≤ 11 12 14 ≥ 15	1.0 (ref) 0.82 (0.51, 1.32) 0.99 (0.66, 1.47) 0.84 (0.49, 1.42) 1.25 (0.77, 2.02)	Age, race, parity, familial history of diabetes, maternal birth weight and activity during pregnancy, pre-pregnancy BMI.
Li et al., 2017, China (13)	1015/6900	2012–2014	13 9–11 12 14 15–18	1.0 (ref) 1.41 (1.06, 1.87) 1.07 (0.89, 1.28) 0.95 (0.79, 1.15) 1.11 (0.88, 1.40)	Age at delivery, education level, occupation, passive smoking exposure during pregnancy, physical activity during pregnancy, number of live births, oral contraceptive use and prepregnancy BMI.
Schoenaker et al., 2017, Australia (14)	357/4749	2000–2012	13 8–11 12 14 15–18	1.0 (ref) 1.51 (1.1, 2.07) 1.07 (0.81, 1.41) 0.96 (0.68, 1.35) 1.13 (0.79, 1.63)	Age, Mother's highest educational qualification, nulliparity, parous status, polycystic ovary syndrome, physical activity and baseline BMI
Shen et al., 2017, USA (15)	168/5914	2007–2012	Normal Early Late	1.0 (ref) 1.75 (1.10, 2.79) 1.04 (0.60, 1.80)	Age at first live birth, race/ethnicity, education, PIR, family history of diabetes mellitus, current marital status, current smoking status, current physical activity level and lifetime greatest BMI

RR, relative risk; CI, confidence interval; N/A, not available; BMI, body mass index; GDM, gestational diabetes mellitus.

### Quality assessment

The results of the quality assessment based on the Newcastle-Ottawa Scale are summarized in Table 2. Two studies received 8 stars, two studies received 7 stars and one study received 6 stars. All of the studies were considered to be high quality. In terms of sample recruitment, 2 cohorts selected population-based samples that were representative of the community [14, 15]. Three cohort recruited samples with specific population (e.g., nurses [11], women who attended prenatal care clinics [12] or women who gave birth at the designated hospital [13]). Bias in assessment of GDM was another common source of bias. The bias in GDM ascertainment is lower in studies that used oral glucose tolerance test for diagnosis of GDM [12, 13] and higher in studies which based on self-reported physician diagnosis [11, 14, 15]. Most studies adjusted for potentially important confounders except for one study, in which prepregnancy body mass index (BMI) was not considered. The follow-up period was over ten years in three studies [11, 12, 14].

**Table 2:** Methodological quality of studies included in the meta-analysis^†^

First Author, Publication Year [reference]	Representativeness of the Exposed Cohort	Selection of the Unexposed Cohort	Ascertainment of Exposure	Outcome of Interest Not Present at Start of Study	Control for Important Factor or Additional Factor^†^	Assessment of Outcome	Follow-up Long Enough for Outcomes to Occur^‡^	Adequacy of Follow-up of Cohorts^§^	Total No. of Stars
Chen, 2016 (11)	—	^*^	^*^	^*^	^*^	—	^*^	^*^	6
Dishi, 2011 (12)	—	^*^	^*^	^*^	^**^	^*^	^*^	^*^	8
Li, 2017 (13)	—	^*^	^*^	^*^	^**^	^*^	—	^*^	7
Schoenaker, 2017 (14)	^*^	^*^	^*^	^*^	^**^	—	^*^	^*^	8
Shen, 2017 (15)	^*^	^*^	^*^	^*^	^**^	—	^*^	^*^	7

^†^A study can be awarded a maximum of 1 star for each item except for the item Control for important factor or additional factor. A maximum of 2 stars could be awarded the item Control for important factor or additional factor. Studies that controlled for age received one star, whereas studies that controlled for other important confounders such as education level, family history of diabetes, physical activity and baseline body mass index (BMI) received an additional star.

^‡^A study with a follow-up duration > 10 y was assigned one star.

^§^A study with a follow-up rate > 70% was assigned one star.

### Early menarche versus “not early” menarche

**Figure 2:**
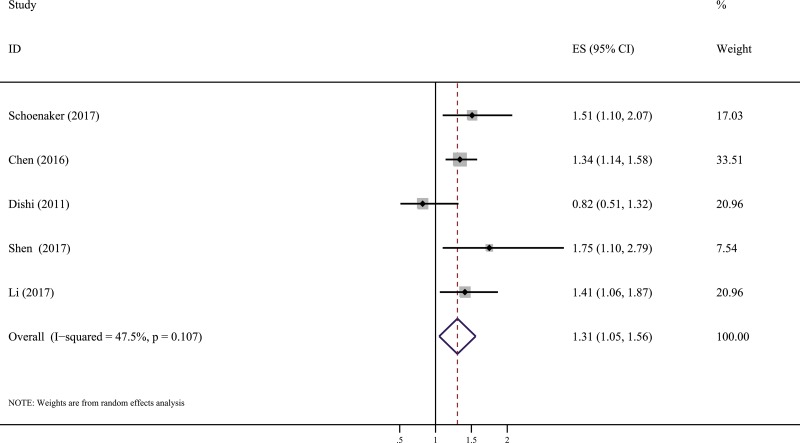
Forest plot (random effects model) of the association between age at menarche and GDM risk with early menarch and “not early” menarche Squares represent the relative risks (RR) for each individual study with the size of the square reflecting the study- specific statistical weight. Horizontal lines indicate 95% confidence intervals (CI). Diamond illustrates the summary RR estimate with its 95% CI.

**Figure 3:**
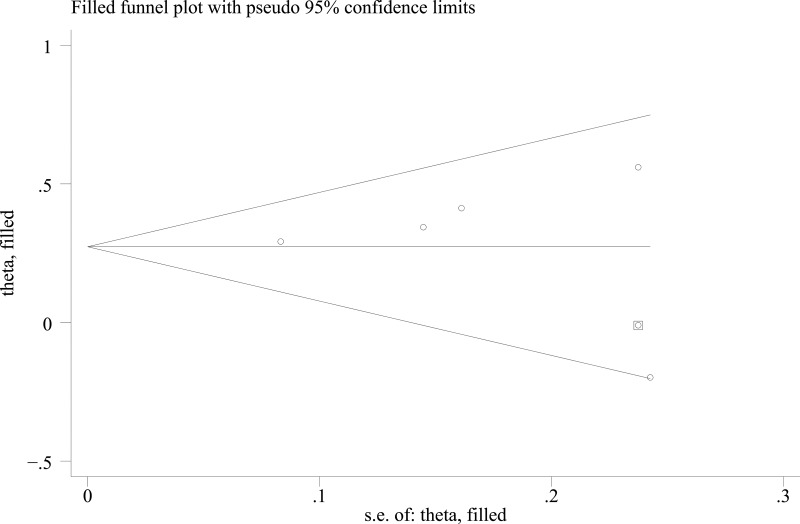
Funnel plot analysis with trim and fill of the association between age at menarche and GDM risk with early menarche and “not early” menarche

Of the 5 studies, 4 studies [11, 13–15] reported significant inverse associations between menarcheal age and the risk of GDM. The early category of age at menarche (< 12 years of age) was compared with “not early” category (at ≥ 13 years of age). The results showed that women in the early menarcheal age group had a higher risk of GDM compared with those in the “not early” menarcheal age group (pooled RR = 1.31, 95% CI: 1.05, 1.56) with moderate heterogeneity by a random effect analysis (*I^2^* = 47.5%, *P* = 0.107) (Figure 2). A symmetrical funnel plot was generated by the trim and fill analysis (Figure 3). When potentially missing studies were added, the results (pooled RR = 1.31; 95% CI: 1.11, 1.54) of this analysis still demonstrated that the correlation between early menarche and GDM risk was significant. It indicated that there was no evidence of publication bias.

### Late menarche versus median menarche

**Figure 4:**
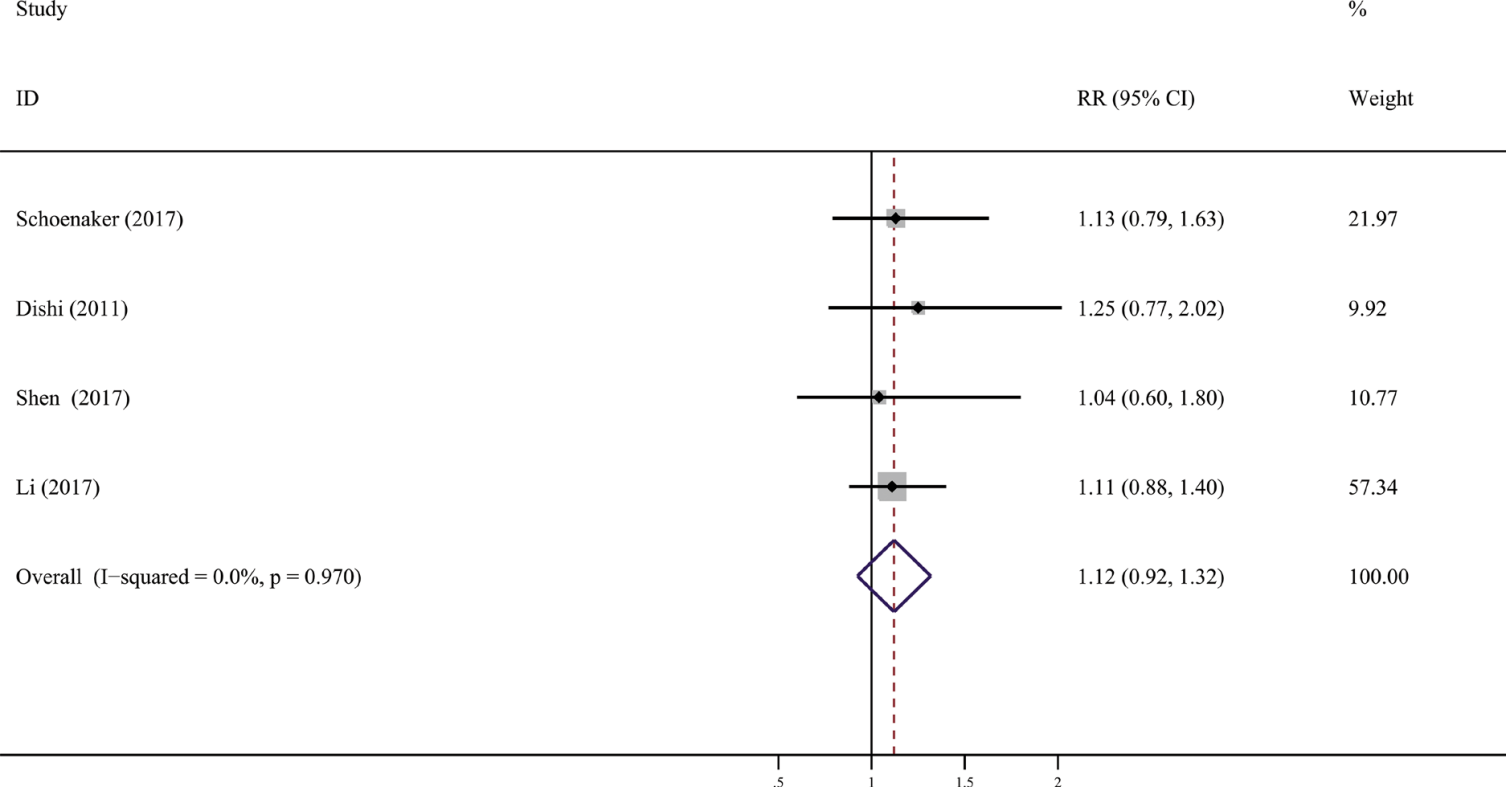
Forest plot (fixed effects model) of the association between age at menarche and GDM risk with late menarche and median menarche Squares represent the relative risks (RR) for each individual study with the size of the square reflecting the study- specific statistical weight. Horizontal lines indicate 95% confidence intervals (CI). Diamond illustrates the summary RR estimate with its 95% CI.

Four studies [12–15] compared the late (at ≥ 15 years of age) with the median menarcheal age group (at 13 years of age). in the risk of GDM. In a fixed effect pooled analysis of the included studies, the summary RR of GDM for the late versus median categories of menarcheal age was 1.12 (95% CI: 0.92, 1.32) (Figure 4). No heterogeneity was found in the study results (*I^2^* = 0%, *P* = 0.97).

### Subgroup analyses

We performed subgroup meta-analyses by study population, duration of follow-up and number of cases and also stratified the meta-analysis by potentially important confounders (Table 3). Results were consistent for studies conducted in America and non-America. The summary estimate was similar for the three studies with ≥ 10 years of follow-up (RR 1.26; 95% CI 1.08, 1.44) and for the two studies with follow-up duration < 10 years (RR 1.47; 95% CI 1.11, 1.84). The pooled RR for two studies with < 400 cases of GDM was not statistically significant (RR 1.18; 95% CI 0.89, 1.42). When stratified by whether considering for potential confounders, we did not found significant differences between estimates adjusted and those not adjusted for specific factors. Furthermore, no evidence of significant heterogeneity between subgroups existed.

**Table 3:** Summary risk estimates of the association between menarcheal age and GDM risk, early versus “not early” category

Subgroup	No. of studies	Summary RR (95% CI)	I^2^	P^*^	P^**^
Study population					
Non-America	2	1.45 (1.14, 1.76)	0	0.76	0.27
America	3	1.25 (1.06, 1.44)	6.3	0.04	
Duration of follow-up					
< 10 y	2	1.47 (1.11, 1.84)	0	0.48	0.306
≥ 10 y	3	1.26 (1.08, 1.44)	67	0.04	
Number of cases					
< 400	3	1.18 (0.89, 1.42)	69.5	0.04	0.33
≥ 400	2	1.36 (1.16, 1.55)	0	0.77	
Adjustment for confounders or important risk factors					
BMI					
Yes	4	1.26 (1.02, 1.50)	59.3	0.06	0.62
No	1	1.30 (1.14, 1.46)	N/A	N/A	
Family history of diabetes					
Yes	3	1.25 (1.06, 1.44)	68.3	0.04	0.27
No	2	1.45 (1.14, 1.76)	0	0.77	

RR: relative risk; CI: confidence interval; BMI, body mass index; N/A: not available. ^*^P value for heterogeneity within each subgroup. ^**^P value for heterogeneity between subgroups.

## DISCUSSION

To the best of our knowledge, this is the first systematic review and meta-analysis to evaluate the effect of menarcheal age on GDM risk. The results of meta-analysis showed that menarcheal age was inversely associated GDM risk, with a 31% higher risk of GDM for women who experienced early menarche (at < 12 years of age). However, there was no obvious protection of late menarche. Inverse associations between age at menarche and the risk of GDM were observed in most sub-groups, but were restricted to studies with < 400 cases of GDM.

The specific mechanisms whereby early menarche increase GDM risk remain poorly understood. There are several possible explanations for the observed association. Accumulating evidence suggests that early menarche is consistently associated with adult obesity [16], which in turn increases the risk of insulin resistance and hyperglycemia. Of the five included studies, three adjusted for prepregnancy BMI [12–14], one adjusted for childhood adiposity [11] and one adjusted for lifetime greatest BMI in which prepregnancy BMI were not available [15]. The association between menarcheal age and GDM risk reported in the three studies [12–14] was substantially attenuated after additional adjustment for prepregnancy BMI, indicating that prepregnancy BMI may involve in the causal pathway of this association. Despite the large attenuation resulting from adjustment for prepregnancy BMI, a strong inverse association between menarcheal age and GDM risk was still apparent, suggesting that other pathways beyond prepregnancy obesity play a role. Earlier age at menarche was also associated with higher estrogen and lower sex hormone-binding globulin (SHBG) levels that persist into adulthood [17, 18]. High plasma estradiol and testosterone, with low SHBG concentrations, have been suggested to be involved in the pathogenesis of type 2 diabetes [19]. Recently, circulating concentration of SHBG was demonstrated to be as a valuable biochemical marker for prediction of risk of GDM [20]. Therefore, it is plausible that hormonal changes could be responsible for the association between menarche and GDM risk.

A strength of this meta-analysis is that the studies included were all prospective design and the results are improbable to be explained by the bias of retrospective studies. Another important strength of this study included a relatively larger sample sizes with at least 2,783 GDM cases (one study used pregnancies instead of cases) and 48,535 subjects. Thus, we had sufficient statistical power to detect putative association between menarcheal age and GDM risk.

Nevertheless, this study also had some limitations. First, age at menarche was self- reported in all studies, which could have caused recall bias. However, previous studies have shown that the recalled age at menarche during adulthood correlated well with the actual childhood data [21]. Second, the diagnosis of GDM was assessed based on self-reported questionnaire in three of the five included studies, which may have potential misclassification bias. Third, the evidence of the associations between age at menarche and GDM risk was limited and quite novel because all of the included studies were published since 2011, and most of them were published in 2017. Fourth, the category of menarcheal age differed between studies, which may contribute to the heterogeneity in results. Finally, the results of subgroup analyses indicated that studies with < 400 cases of GDM showed no significant associations between age at menarche and GDM risk. Further larger prospective studies are warranted to identify our findings.

In conclusion, the current study provides evidence that younger age at menarche is an independent risk factor for GDM. The findings may be a useful tool for future intervention strategies to identify girls who are at increased risk of the development of GDM in adulthood.

## MATERIALS AND METHODS

### Search strategy and study selection

A comprehensive electronic search was performed using the Medline (PubMed), Embase, Web of Knowledge databases and the Cochrane library to the end of May 2017. The search terms used were (menarche OR menstruation) AND (gestational diabetes OR gestational diabetes mellitus OR (hyperglycaemia AND pregnancy)). Manual search was also conducted through reference lists of reviews and relevant studies. No limit was placed on language.

Studies were included in this review if they met all the following criteria. (1) The study had to be a case-control, cohort or cross-sectional study design. (2) The population included were adult women. (3) The study evaluated the association between menarcheal age and GDM risk and provided odds ratio (OR), relative risk (RR) or hazard ratio (HR) estimates with 95% confidence intervals (CI) for this association. Studies conducted in animals were excluded.

### Data extraction and quality assessment

Two researchers independently extracted data, including author list, year of publication, study region, study design, study sample size (number of cases and cohort size), follow-up period for cohort studies, menarcheal age categories and risk estimates with their corresponding CI (if there were multiple estimates, we extracted the estimate which adjusted for the most covariates), and factors adjusted in the analysis. Disagreements were resolved by discussion.

The quality of the included studies was evaluated using the Newcastle–Ottawa scale for cohort studies [22]. The scale consists of 8 items, based on the selection (representativeness of the exposed cohort, selection of the unexposed cohort, ascertainment of exposure, demonstration that the outcome of interest was not present at the start of the study), comparability (Control for important factor or additional factor), and the outcome (outcome assessment, long enough follow-up, adequacy of follow-up of cohorts). Two independent investigators reviewed each of the studies, giving a maximum of 9 stars to any individual study. Studies with stars more than or equal to 6 was defined as a high quality study.

### Data analysis

The early category of age at menarche (< 12 years of age) was compared with “not early” category (at ≥ 13 years of age). In addition, we also compared the late (at ≥ 15 years of age) with the median menarcheal age group (at 13 years of age). For the meta-analysis, we assumed that estimates of risk, rate or hazard ratios from prospective studies were all valid estimates of the relative risk and we refer to RR for all types of measures for simplicity.

*I^2^* statistic was used to quantify the possible heterogeneity across the studies. We considered that *I^2^* values of 50% or less indicated low heterogeneity and *I^2^* ≥ 50% indicated substantial heterogeneity [23]. We used a random effects model to calculate summary RRs and 95% CIs for the earliest versus “not early” category of age at menarche and a fixed effects model for the oldest versus the median menarcheal age group. We assessed potential sources of heterogeneity by conducting subgroup analyses, which were carried out based on study population, duration of follow-up and number of cases. We also stratified the meta-analysis by potentially important confounders. We used the trim and fill method to assess publication bias. All analyses were conducted with Stata, version 12.0, software (StataCorp LP, College Station, Texas).
